# The Role of Insulin and Insulin-Like Growth Factor-1/FoxO-Mediated Transcription for the Pathogenesis of Obesity-Associated Dementia

**DOI:** 10.1155/2012/384094

**Published:** 2012-05-13

**Authors:** Lorna Moll, Markus Schubert

**Affiliations:** Center for Endocrinology, Diabetes and Preventive Medicine, University of Cologne, CMMC Building 66, 5.012, Robert-Koch-Straße 21, 50931 Cologne, Germany

## Abstract

Epidemiological studies suggest that being obese in midlife is a risk factor for cognitive decline and dementia in later life. Hyperinsulinemia is one of the most frequent endocrine features in overweight people which results in insulin desensitization. Thus, chronically high insulin levels have been identified as risk factor for dementia. Accordingly, chronically high insulin levels might be harmful for brain function. Furthermore, insulin and IGF-1-induced signaling is reduced in the brains of patients suffering from Alzheimer's disease (AD). Interestingly, studies in rodents suggest that reduced insulin receptor (IR) and insulin-like growth factor-1 receptor (IGF-1R) signaling decrease AD pathology, that is, **β**-amyloid toxicity. Data obtained in *C. elegans* indicate that the beneficial effect mediated via reduced IR/IGF-1R signaling might partially be induced via the forkhead-box O transcription factors (FoxO). In the mammalian brain, there are FoxO1, FoxO3a, and FoxO6 expressed. Surprisingly, high-fat diet specifically reduces the expression of FoxO3a and FoxO6 suggesting that IR/IGF-1 → FoxO-mediated transcription is involved in the pathogenesis of obesity-associated cognitive impairment. Therefore, the function of FoxO1 and FoxO3a has been investigated in animal models of Alzheimer's disease in detail. The current paper focuses on the role of IR/IGF-1 signaling and IR/IGF-1 → FoxO-mediated transcription for the pathogenesis of obesity-associated dementia.

## 1. Introduction

Obesity is characterized by a body mass index (BMI) of over 30 kg/m^2^. The prevalence of obesity will rise to approximately 700 million people worldwide in 2015 [1]. Furthermore, midlife overweight and obesity might increase the risk for dementia during aging [2–4]. Hence, the role of obesity or overweight status in the development of cognitive decline or dementia is a major health concern and possibly associated with enormous health care costs. Prospective investigations on the role of BMI for the development of dementia did not provide a conclusive picture, yet. Some studies report no association or even decreased BMI to be associated with dementia or Alzheimer's disease [5, 6], and others suggested higher BMI to be a risk factor for dementia [7] or that overweight in middle age is associated with dementia decades later [8, 9]. It seems to be difficult to estimate the exact role of obesity itself for the initiation or progress of cognitive impairment. Furthermore, obesity is associated with a variety of cardiovascular risk factors influencing long-term cognitive performance. Moreover, lower cognitive abilities are a risk factor for obesity, but on the other hand, dementia in later life might be associated with lower BMI. Thus, it might well be that obesity in younger or midlife is a risk factor for dementia, and dementia is causing weight loss and cachexia on the long run. Taken together, cognitive performance might influence the pathogenesis of obesity and being overweight the development of cognitive impairment, dementia, and neurodegeneration. This interrelationship between body weight and cognitive function implicates the need for lifetime studies and standardized tests to identify cause or consequences of obesity-associated dementia. The complex interplay might at least partially explain the different results obtained by different studies. However, there is growing evidence that disturbed metabolic signals in obesity or type 2 diabetes feedback to the central nervous system (CNS) influencing brain function and possibly the pathogenesis of dementia or cognitive decline.

Recently, insulin and insulin-like growth factors (IGFs) have been suggested as important modifiers for the pathogenesis of neurodegenerative diseases, providing a link between obesity, type 2 diabetes (T2D), and cognitive impairment or even the pathogenesis of Alzheimer's disease. An important key mediator of insulin and IGF-1-mediated effects are the forkhead box O (FoxO) transcription factors. These transcription factors are involved in the neuronal proliferation, differentiation, stress response, and *β* amyloid detoxification.

The current review discusses the role of insulin and insulin-like growth factor-1/FoxO-mediated transcription for the pathogenesis of obesity-associated dementia from model organisms to humans.

## 2. Obesity and Dementia

As mentioned above, there might be a complex interplay between cognition and metabolic signals between the peripheral blood and the CNS. Obesity is associated with a whole variety of metabolic signals feeding back to the brain for example, leptin, insulin, or different cytokines. Furthermore, timing of “metabolic injuries” might be crucial for cognitive function during later life. Thus, it is not surprising that epidemiological studies show different results depending on the study collective, duration of study, phase of life investigated, and comorbidities (e.g., T2D).

In detail, Stewart and coworkers showed that in a prospective, population-based study of Japanese American men over a 32-year period that (dementia-associated) weight loss begins before the onset of the clinical syndrome and accelerates by the time of diagnosis [6]. Thus, weight loss is part of the clinical presentation of patients suffering from dementia, for example, AD. Another study showed a link between an increased BMI and the risk to develop Alzheimer's dementia in elderly people 10 years before the onset of symptoms, suggesting that the period in life of being overweight might be an important issue [7].

Interestingly, there are several studies supporting the hypothesis that obesity in midlife plays a role in the development of dementia in later life [8, 9].

The possible role of the surveillance periods of exposure to overweight for cognitive function during life has recently been reviewed by Elias and coworkers [10]. In addition, the Swedish Adoption/Twin Study of Aging (SATSA) focused on the relation of BMI and cognitive decline over a time period of over 40 years. This study showed an association of higher BMI in midlife and cognitive decline in males and females [11]. Furthermore, the Finnish Twin Cohort Study revealed that the BMI in midlife as well as cardiovascular risk factors are associated with reduced cognitive abilities during aging [12].

Taken together, low BMI and weight loss seem to be the first clinical manifestation of neurodegeneration even before the onset of perceivable cognitive impairment. However, even still heterogeneous, recent data suggest that certain periods of exposure to obesity during life might be crucial for cognitive function during aging.

## 3. Hyperinsulinemia and Dementia

Chronic hyperglycemia impairs cerebral blood flow [13] as well as central glucose utilization [14]. Interestingly, central insulin ameliorates cognitive function, and acute central insulin administration leads to improved spatial memory in mice [14]. Accordingly, intranasal application leads to increased verbal memory in humans [15]. In the Nurses' Health Study (NHS), nondiabetic women with higher fasting insulin levels tended to have lower performance on global and verbal cognition. Furthermore, higher levels of fasting insulin were associated with faster rates of cognitive decline [16]. However, insulin levels were relatively low in this study indicating a potential role of even modestly elevated insulin concentrations. In the Physicians' Health Study, higher late-life fasting insulin levels among nondiabetic men were associated with a greater subsequent decline in general cognition [17]. Taken together, insulin in the short run might improve cognition, but chronically elevated insulin levels are associated with faster cognitive decline during aging. Thus, alterations of insulin levels and insulin-mediated signals/transcription might be an interesting candidate to explain the association of obesity and dementia.

## 4. Insulin/Insulin-Like Growth Factor-1 Signaling

Insulin receptors (IRs) are present in the so-called classic insulin responsive tissues such as muscle, fat, or liver and nonclassic tissues such as brain, endothelial cells, or gonadal cells. In 1978, Havrankova et al. demonstrated for the first time the localization of IRs in the CNS, an organ classically considered as an insulin-insensitive tissue. Moreover, insulin enters the CNS across the blood brain barrier through an active transport mechanism [18–21]. The localization of insulin receptors in the CNS was assessed by various techniques, including *in vitro* binding studies [22], *in vivo* and *in vitro* autoradiography and computerized densitometry [21–25] and immunocytochemistry [26]. According to these studies, insulin receptors are widely distributed in the brain with highest concentrations in the olfactory bulb, hypothalamus, cerebral cortex, and hippocampus.

**Figure 1:**
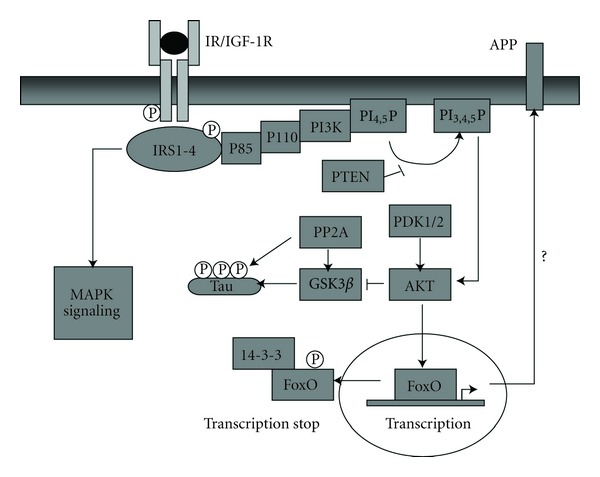
Central IR/IGF-1 signaling. Binding of insulin and IGF-1 to their receptors leads to autophosphorylation of the *β*-subunits of the IR or IGF-1R, recruitment of IRS-1/-2, and subsequently activation of mainly two pathways the PI3 kinase pathway and the MAP kinase cascade. The PI3 kinase pathway activates Akt which inhibits GSK-3*β*. Akt-mediated FoxO1 phosphorylation results in binding of the regulatory protein 14-3-3 and nuclear exclusion of FoxO1. Abbreviations: IR, insulin receptor; IGF-1R, insulin-like growth factor-1 receptor; IRS, insulin receptor substrate; PI3K, PI3 kinase; FoxO1, forkhead-box protein O1; PDK, phosphatidylinositide-dependent kinase; p110/p85, catalytic/regulatory subunit of PI3K; PI_3,4_P, phosphatidylinositide  _3,4_-diphosphate; PI_3,4,5_P, phosphatidylinositide 3,4,5-triphosphate; PTEN, phosphatase and tensin homolog deleted on chromosome ten; 14-3-3, regulatory protein 14-3-3; PP2A, protein phosphatase 2A.

The insulin and IGF-1 receptor (IR/IGF-1R) are tyrosine kinases which consist of a membrane bound domain with tyrosine kinase activity. This tyrosine kinase phosphorylates tyrosine residues of downstream signaling proteins like insulin receptor substrates (IRSs). The IR and IGF-1R have a heterotetrameric structure with extracellular localized *α*-subunits and membrane bound *β*-subunits. These *β*-subunits contain ATP-binding motifs, autophosphorylation sites, and tyrosine protein kinase activity activated after binding of insulin or IGF-1 to the receptor [27–29]. Binding of the ligand results in conformational change of the receptor and induces autophosphorylation followed by recruitment of IRS proteins which get thereby tyrosine phosphorylated. The IRS protein family consists of four members, IRS-1 to 4 [30–32]. These IRS proteins consist of an pleckstrin homology (PH) domain located at the N-terminus, a phosphotyrosine-binding (PTB) domain and a C-terminus with multiple tyrosine phosphorylation sites. These phosphotyrosine motifs of the IRS proteins are binding sites for Src homology (SH)2 domain-containing proteins [33]. Furthermore, the PH domain interacts with phosphoinositides, while the PTB domain binds to phosphotyrosine residues of, for example, the IR and IGF-1R [34–36]. Insulin induces tyrosine and serine phosphorylation of IRS-1 which leads to positive or negative regulation of IRS-1 and the downstream signaling pathway [37–39]. The mammalian phosphatidylinositide (PI) 3-kinase family consists of classes I to III, and class I is subdivided into classes Ia and Ib [40]. PI3K, a class Ia kinase, induces phosphorylation of the 3′ hydroxyl position of phosphatidyl-myo-inositol lipids [41]. The PI3K shows a heterodimeric structure with a catalytic 110 kDa subunit which is noncovalently bound to a 50-, 55-, or 85 kDa regulatory subunit. After binding and activation of IRS to the IR or IGF-1R, the PI3K is recruited to the membrane using the p85 regulatory subunit. Additionally, the growth factor receptor binding protein (GRB)-2 and the SH2-phosphatase (SHP) 2 are recruited after activation of the IR/IGF-1R signaling pathway. Activation of the PI3K leads to phosphorylation of phosphatidylinositide diphosphate (PI_4,5_P) to generate phosphatidylinositide triphosphate (PI_3,4,5_P). The phosphorylation of PI_4,5_P is reduced via PTEN (phosphatase and tensin homolog deleted on chromosome ten) action. Following this step, the downstream signaling proteins like phosphoinositide-dependent protein kinase (PDK) and protein kinase B (PKB, AKT) are activated. PDK has two isoforms, PDK-1 and PDK-2. PDK-1 phosphorylates AKT at Thr308 [42–44]. AKT is a serine/threonine kinase with a size of 57 kDa. AKT occurs in three isoforms, AKT-1 to AKT-3. The structure of AKT consists of a PH domain, a kinase domain, and an N- and C-terminal regulatory subunit [45]. In addition to the PI3K pathway, insulin and IGF-1 activate the MAP kinase (MAPK, mitogen-activated protein kinase) signaling cascade (Figure 1) [36, 46, 47].

## 5. Forkhead-Box O Transcription Factors

FoxOs differ in their expression pattern. FoxO1 and FoxO3a are ubiquitously expressed. In contrast, FoxO6 only occurs in the brain, whereas FoxO4 has not been found in the brain so far [48, 49]. FoxO1 is predominantly expressed in the dentate gyrus, striatum, and ventral hippocampus, while FoxO3a mainly occurs in the cerebellum, cortex, and hippocampus. FoxO6 is found in the hippocampus, amygdala, and cingulate cortex of the adult murine brain [50, 51].

Activated AKT phosphorylates forkhead-box O transcription factors which leads to binding of 14-3-3 inducing their nuclear exclusion. This inactivates FoxO-mediated transcription which regulates apoptosis, growth, metabolism, and cellular differentiation under active conditions [52].

**Figure 2:**
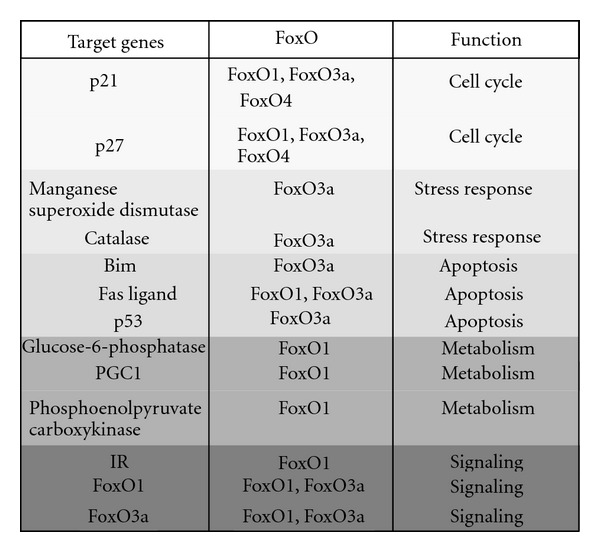
Function and targets genes of FoxO transcription factors. Abbreviations: IR, insulin receptor; PGC1, peroxisome-proliferative-activated receptor-*γ* (PPAR*γ*) coactivator 1; BIM, Bcl2-interacting mediator of cell death; p21, cyclin-dependent kinase inhibitor 1 (review in [58]).

**Figure 3:**
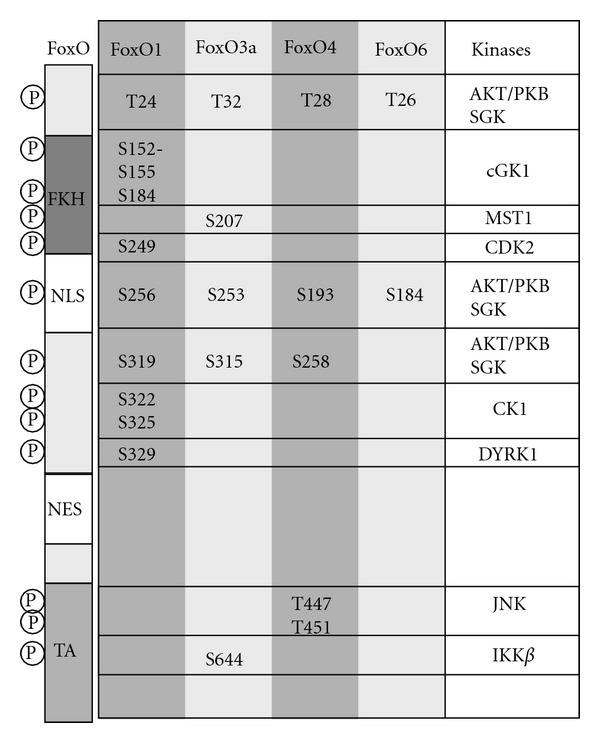
FoxO phosphorylation sites regulated via different kinases. Abbreviations: PKB, protein kinase B/Akt; SGK, serum-and glucocorticoid-induced protein kinase; cGK, cGMP-dependent protein kinase type 1; MST1, mammalian sterile 20-like 1 kinase; CDK 2, cyclin-dependent kinase 2; CK1, casein kinase 1; DYRK, dual specificity tyrosine-phosphorylation-regulated kinase; JNK, c-Jun N-terminal kinase; IKK, I*κ*B-kinase, NLS, nuclear localization signal; NES, nuclear export signal; FKH, forkhead domain; TA, transactivation domain.

The mammalian FoxO transcription factor family contains 4 proteins: FoxO1, FoxO3a, FoxO4, and FoxO6. These proteins share a conserved DNA binding domain, the forkhead domain (FKHR) binding to a consensus FoxO-recognized element (FRE) sequence of the target gene (G/C)(T/A)AA(C/T)AA [48, 53, 54]. Target genes of FoxO-mediated transcription are, for example, Fas ligand (FasL), p27^KIP1^ [55, 56], and manganese superoxide dismutase (MnSOD) [57] (Figures 2 and 3).

FoxO-mediated transcription is regulated via posttranslational modifications. One major modification is the phosphorylation of different sites within FoxOs. Upon activation, AKT phosphorylates FoxO1 at Thr24, Ser256, and Ser319 [53, 59–62]. FoxO3a becomes phosphorylated at Thr32, Ser253, and Ser315 via AKT [63]. Furthermore, FoxOs are phosphorylated via different kinases depending on the stimulus (review in [64]). Other posttranslational modifications are ubiquitination. FoxO1 is ubiquitinated via Skp2, the substrate-binding component of the Skp1/culin 1/F-box protein (SCF^Skp2^) E3 ligase complex. This ubiquitination occurs after phosphorylation of FoxO1 at Ser256 via AKT [65–68]. FoxO1 and FoxO3a are polyubiquitinated, while FoxO4 is monoubiquitinated for degradation [69]. Additionally, FoxO transcription factors are methylated, for example, FoxO1 gets methylated at Arg248 and Arg250. These sites are located in the AKT phosphorylation motif. This methylation is promoted by the protein arginine N-terminal methyltransferase 1 (PRMT1) protecting FoxO1 from phosphorylation via AKT, translocation out of the nucleus, and degradation [70]. Furthermore, FoxOs are acetylated via CBP and p300 with their interacting proteins like CBP- and p300-associated factor (PCAF) [71]. The acetylation of FoxOs decreases DNA binding and promotes phosphorylation of FoxO via AKT which inactivates FoxOs [72, 73]. Deacetylation of FoxOs is induced by silent information regulator 1 (SIRT1), a nicotinamide-adenine-dinucleotide- (NAD-) dependent histone deacetylase [74, 75].

FoxO-mediated transcription is involved in several processes. One of them is controlling cell cycle arrest via regulation of transcription of, for example, the cyclin-dependent kinase inhibitor p27 (review in [76]). Under conditions of growth factor deprivation, the IR/IGF-1R signaling pathway is inactive, and FoxOs are active inducing cell cycle arrest and quiescence to promote survival [57]. Additionally, FoxOs are involved in oxidative stress response (Figures 2 and 3). To counteract reactive oxygen species (ROS) produced during oxidative stress, FoxOs increase expression of antioxidant enzymes like MnSOD [57].

## 6. Expression of FoxO during Aging and High-Fat Diet

FoxO transcription factors show a distinct temporal and spatial expression pattern at least in the murine brain. The analysis of FoxO expression in different regions of mouse brain up to 100 weeks of age revealed that FoxO1 is predominantly expressed in the hippocampus compared to overall expression in the whole brain, whereas FoxO3a showed its highest expression in the cerebellum [51]. Furthermore, expression of FoxOs in C57BL/6 mice strongly respond to a high-fat diet (HFD) at least if fed over 46 weeks. Western blot analysis of cortex lysates of these HFD mice revealed increased phosphorylation of AKT at Ser473 compared to mice on STD (standard diet) indicating increased IR/IGF-1 signaling in these mice. Surprisingly, FoxO1 mRNA levels were slightly increased in the CNS of HFD mice, wheras FoxO3a mRNA levels were significantly decreased in the cerebellum, frontal, parietal, and occipital cortex. Even more strikingly, the expression of FoxO6 was upto 80% decreased in all analyzed brain regions [51]. In line with these *in vivo* data, SH-SY5Y human neuroblastoma cells stably overexpressing IRS-2 showed increased phosphorylation of AKT at Ser473 and analysis of mRNA levels of FoxO3a revealed significantly reduced expression as observed in the CNS of mice fed a HFD indicating that chronically elevated IR/IGF-1R signaling in neurons leads to downregulation of FoxO3a *in vivo* and *in vitro* [51]. The exact molecular mechanism how IR/IGF-1R signaling cascade regulates expression of the different FoxOs is still under investigation. However, data obtained in cell lines might not exactly reflect the *in vivo* situation. Thus, hyperinsulinemia in mice fed a HFD over a long period induces decreased expression of FoxO transcription factors, for example, FoxO3a and FoxO6, suggesting that disturbance of FoxO-mediated transcription downstream of the insulin signaling pathway might be involved in the pathogenesis of obesity-associated cognitive dysfunction. The hypothesis is supported by animal experiments indicating a role for IRS-2-mediated signals for memory formation [77]. Mice harboring a brain-specific deletion of IRS-2 showed enhanced spatial working memory. Taken into account that decrease in IRS-2 signaling induces FoxO-mediated transcription, the assumption that decreased FoxO expression observed in HFD mice is involved in the pathogenesis of obesity-associated cognitive impairment seems reasonable or even likely. There is growing evidence that at least in AD IR/IGF-1 signaling is disturbed. Thus, IR/IGF-1-regulated transcription might be a part of the pathogenesis of at least AD. However, it is still unclear whether the changes in neuronal IR/IGF-1 signaling is cause or counterregulation in response to neurodegeneration.

## 7. Alzheimer's Disease

Alzheimer's disease is a chronic and progressive neurodegenerative disease. It is the most common form of dementia leading to cognitive decline and death [78, 79]. Characteristics of AD are neurofibrillary tangles (NTFs) and *β* amyloid plaques. NTFs consist of hyperphosphorylated (abnormal high phosphorylation) tau proteins, and *β* amyloid plaques contain aggregated amyloid-*β* (A*β*) peptides [80, 81]. It is hypothesized that the accumulation of A*β* is the leading cause for neurodegeneration in the progression of AD [80].

Several clinical studies showed that the IR/IGF-1R signaling is impaired in the central nervous system (CNS) of patients suffering from AD [82–84]. The expression of the IR and the IGF-1R was reduced in brains of AD patients [84, 85], whereas increased IGF-1 serum levels were detected [85, 86]. Additionally, the expression levels of IRS-1 and IRS-2 were reduced, and the inhibitory serine phosphorylation of IRS-1 at Ser312 and Ser616 was increased in AD brains. These findings indicate AD to be a brain type diabetes [87].

Tau predominantly occurs in the axons of neurons [88] and is less present in dendrites [89]. Tau might be involved in stabilization of microtubules and regulation of axonal transport [90]. It contains an N-terminal projection domain and a short tail sequence. The C-terminal domain consists of microtubule-binding (MTB) repeats. Tau gets phosphorylated at different sites by a variety of kinases. GSK3*β* predominantly phosphorylates tau and is regulated via the IR/IGF-1R signaling pathway. Activated AKT phosphorylates Ser9 of GSK3*β* to inhibit its action. An important phosphatase of tau is PP2A (protein posphatase 2A) [91]. This phosphatase is also regulated via the IR/IGF-1R signaling cascade indicating that insulin and IGF-1 action promotes both phosphorylation and dephosphorylation leading to equilibrium of tau phosphorylation at least under certain circumstances [91, 92].

A*β* peptides, the main component of amyloid plaques, are produced via proteolytic cleavage of the amyloid precursor protein (APP). APP belongs to the type-1 integral membrane protein family [93–96]. The APP gene is localized on chromosome 21, therefore patients suffering from trisomy 21 present an increased risk for AD. Hence, increased APP expression results in Alzheimer-like pathology [97, 98]. Furthermore, mutations in the APP gene itself like the Swedish mutation (APPsw) or mutations in presenilin 1 and presenilin 2 which are involved in proteolytic cleavage of APP lead to familial early-onset AD (FAD) [99–107].

**Figure 4:**
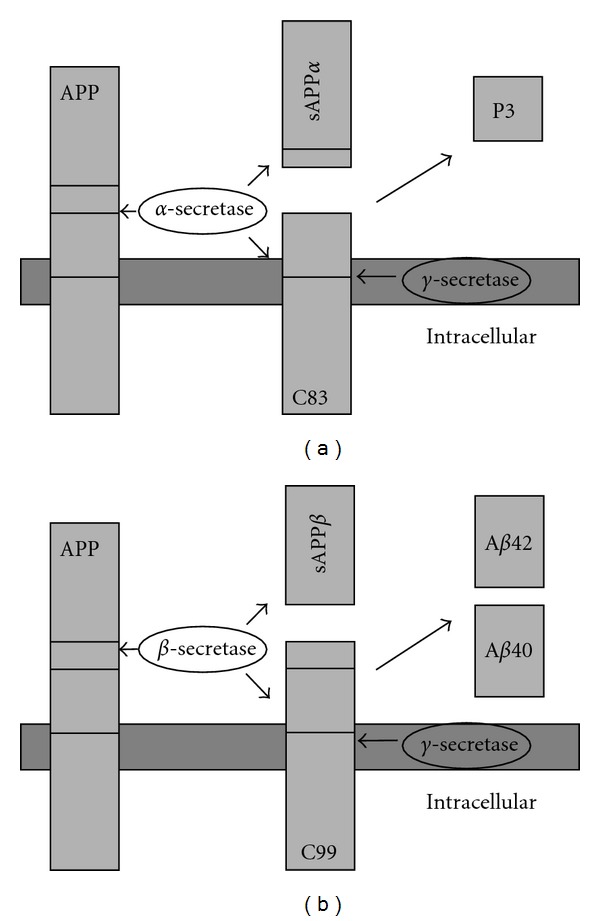
APP cleavage. (a) Nonamyloidogenic pathway: APP is cleaved by *α*-secretase leading to a membrane-bound C83 (*α* c-terminal fragment, *α*CTF) and the soluble APPs*α*; (b) amyloidogenic pathway: APP is cleaved via the *β*-secretase (BACE-1) and *γ*-secretase (presenilin complex). *β*- and subsequent *γ*-cleavage of APP leads to generation of *β*-amyloid_40/42_(A*β*). Abbreviations: APP, amyloid precursor protein; A*β*, *β*-amyloid; BACE, *β*-site cleavage enzyme.

Different APP splicing variants with distinct molecular weight are generated *in vivo*. APP containing 751 or 770 amino acids (APP751 and APP770) is expressed in nonneuronal tissue, while APP695 is predominantly found in neurons [108]. The function of APP and APP-like protein (APLP) is not well understood. These proteins might be involved in apoptosis, axonal transport, and cell adhesion. APP and APLP are expressed in nearly all vertebrates and invertebrates [109–111]. The structure of APP consists of an N-terminal extracellular and a C-terminal domain located in the cytoplasm. Proteolytic cleavage of APP is promoted via the *β*-secretase BACE-1 (*β*-site APP cleaving enzyme) which leads to the “amyloidogenic pathway” of APP cleavage. APP is cleaved at Asp^+1^ of the N-terminus via BACE-1 which leads to the generation of soluble APPs*β* and the C-terminal fragment C99 (Figure 4). C99 is cleaved via the *γ*-secretase, a complex formed by presenilin, nicastrin, Aph-1, and Pen-2. This cleavage results in the production of A*β* (4 kDa) and the APP intracellular domain (AICD) with a size of 6 kDa. A*β*-peptides are mainly found in two variants which are distinguishable because of their size. A*β*40 ends at residue 40 and A*β*42 ends at residue 42. Predominantly, the A*β*42 is susceptible to aggregate and forms neurotoxic oligomers. In contrast, the “nonamyloidogenic pathway” starts with cleavage of APP via the *α*-secretase ADAM10 (a disintegrin and metalloproteinase-like 10) or TACE (tumor necrosis factor-alpha convertase) which leads to the generation of the soluble APPs*α* and the C-terminal fragment C83. The decision whether the amyloidogenic or nonamyloidogenic cleavage pathway is induced depends on the competition of the *α*- and *β*-secretase [107, 110]. Up to 90% of all A*β*-peptides in brains of healthy people are A*β*40, whereas A*β*42 is less produced with about 5 to 10% [112]. The accumulation of A*β*42 is a major step in the formation of A*β* oligomers amyloid plaques [113]. A*β* oligomers show an increased cytotoxic effect compared to mature A*β* fibrils [114–116]. A*β*-derived diffusible ligands (ADDLs) are aggregates with a size of about 17 to 42 kDa and present no fibrillar structure but are neurotoxic [117–119], and the concentrations of ADDLs correlate to cognitive impairment in AD [120].

The exact mechanism how A*β* facilitates its neurotoxic effect is not completely understood yet, but toxicity might be induced via generation of ion channels, membrane disruption, oxidative stress, induction of apoptosis, and inflammation [121–124].

## 8. Insulin/IGF-1 Signaling and FoxO-Mediated Transcription in the Pathogenesis of Alzheimer's Disease

In cultured human neurons, Hong et al. [125] showed that glycogen synthase kinase-3 (GSK-3) phosphorylates the neuronal protein tau. Hyperphosphorylated tau is the major component of paired helical filaments in neurofibrillary lesions associated with Alzheimer's disease. Hyperphosphorylation reduces the affinity of tau for microtubules and is thought to be a critical event in the pathogenesis of tauopathies. Insulin and IGF-1 have been shown to reduce the phosphorylation of tau protein by inhibiting activity of GSK-3. *In vivo* IRS-2-deficient mice, a model of insulin resistance and type 2 diabetes, displayed tau-hyperphosphorylation and developed intracellular deposits of hyperphosphorylated tau during aging [126] suggesting a critical role for IR/IGF-1 signaling in regulation of tau phosphorylation *in vivo*.

Previous studies suggest that insulin and IGF-1 support neuronal survival *in vitro*. In particular, insulin/IGF-1 strongly activates AKT/PKB to promote BAD phosphorylation and its association with 14-3-3, which releases Bcl-2 to inhibit apoptosis [127]. Furthermore, IR/IGF-1-receptor signal transduction regulates the processing and secretion of APP [128, 129], and Xie et al. [129] have demonstrated that *β*-amyloid peptides compete for insulin's binding to the IR. As mentioned above, IR/IGF-1R is reduced in AD brains strongly suggesting a role for IR/IGF-1 signaling in the pathogenesis of AD. This is supported by several animal models. For example, IGF-1-deficient mice presented increased tau phosphorylation at Ser396 and Ser202 [130]. The brain-specific knockout of the IR (NIRKO) showed highly phosphorylated tau at Thr231 [131]. IRS-2-deficient mice displayed “hyperphosphorylation” at Ser202 [126]. Thus, genetically induced IGF-1 or insulin resistance in mice induces tau hyperphosphorylation, indicating that at least the tau part of AD pathology is enhanced by insulin resistance *in vivo*.

In contrast, animal experiments suggest a different role of IR/IGF-1 signaling for the *β* amyloid pathology in AD. Tg2576 mice express the human-derived APP harboring the Swedish mutation (APPsw) inducing increased A*β* burden and AD-like pathology [107, 132–135]. Mice with a neuron-specific IGF-1R deletion (nIGF-1R^−/−^) or IRS-2 knockout (IRS-2^−/−^) crossed with Tg2576 mice were protected from premature death and showed decreased A*β* accumulation [136]. Interestingly, neuron-specific deletion of the IR (nIR^−/−^) in a Tg2576 background showed decrease in A*β* burden but displayed no survival benefit compared to Tg2576 mice [137].

A rather interesting study investigated the role of partial IGF-1 resistance in an AD mouse model overexpressing APPsw and the human presenilin-1 ΔE9 variant under control of the prion promoter [138]. These AD mice were crossed with mice heterozygote for the IGF-1R (Igf1r^+/−^). These APPsw and presenilin-1 ΔE9/Igf1r^+/−^ mice showed an increased assembly of A*β* into densely packed, fibrillar structures [139]. This so-called hyperaggregation suggests an active aggregation of highly toxic A*β* oligomers to densely packed aggregates which are less neurotoxic. In summary, this study suggests that partial IGF-1 resistance protects the brain from neurotoxicity mediated via A*β* oligomers [139].

In *Caenorhabditis elegans,* the knockdown of DAF-2, the ortholog of mammalian IR and IGF-1R using siRNA (small interference RNA) has been shown to reduce A*β*42 toxicity [140]. This reduced toxicity was caused via the downstream transcription factor DAF-16 (Abnormal dauer formation 16), the ortholog of the mammalian FoxO1 and FoxO3a, and HSF-1 (heat shock transcription factor-1) [140–142]. HSF-1 induces disaggregation of the toxic A*β* oligomers and degradation. In case this mechanism is oversaturated, DAF-16 promotes the formation of hyperaggregated A*β*-peptides which leads to aggregates with high-molecular weight and less toxicity [140].

Recently, two mouse models expressing neuron-specifically a dominant negative or constitutive active form of FoxO1 (FoxO1DN and FoxO1ADA), have been published. The first mutant, FoxO1ADA, is nuclear expressed due to mutation of T24 and S316 to A as well as a mutation of S256 to D and acts constitutively active. The second FoxO1 mutant is transactivation domain deleted (FoxO1DN) and exerts a dominant negative effect. These mice were crossed with Tg2576 mice and analyzed in respect to survival, APP processing, and A*β* aggregation. FoxO1DN mice in a Tg2576 background showed no differences in survival up to 60 weeks of age compared to Tg2576 mice in both genders. Interestingly, FoxO1ADA mice which mimic the situation of an inactive IR/IGF-1R signaling pathway presented no differences in A*β* burden. However, these mice showed an increased mortality compared to Tg2576 mice for a yet unknown reason [137]. Thus, in mammals FoxO1-mediated transcription does not explain the beneficial effects mediated via the IR/IGF-1 pathway.

An alternative downstream candidate of the IR/IGF-1R signaling pathway is FoxO3a. Caloric restriction activates the IR signaling pathway resulting in phosphorylation of FoxO3a and nuclear exclusion [143]. A recent study showed that this inactivation of FoxO3a leads to attenuation of AD pathology and preservation of spatial memory in Tg2576 mice. Accordingly, *in vitro* studies using primary corticohippocampal Tg2567 neuron cultures expressing a constitutive active FoxO3a revealed increased A*β*-peptide production. In addition, FoxO3a is deacetylated by SIRT1 which might result in inhibition of FoxO3a-mediated transcription during caloric restriction. This leads to reduced Rho-associated protein kinase-1 (ROCK1) gene expression followed by activation of the nonamyloidogenic processing of APP and decreased levels of A*β*-peptides [143]. Thus, animal experiments support the hypothesis that increased FoxO-mediated transcription does not protect but rather increase amyloid pathology.

The clearance of A*β* from the brain is facilitated via different mechanisms: (i) transport across the blood brain barrier (BBB), (ii) phagocytosis by microglia, and (iii) enzymatic degradation. Transport across the BBB is promoted via A*β* binding to the low-density lipoprotein receptor-related protein (LRP) directly or via its associates to LRP in complex with APOE (apolipoprotein E) and/or *α*2-macroglobulin (*α*2M). After crossing the BBB A*β*-peptides are delivered to peripheral tissues, for example, liver for degradation [144]. Previous studies proposed that IGF-1 plays an important role for degradation and clearance of A*β*. This was supported by the finding that Tg2576 mice have decreased IGF-1 levels in comparison to wild-type animals and that the treatment with IGF-1 causes an increased transport of A*β* out of the brain [145]. Thus, there might be a different role for IGF-1R signaling in neurons and serum IGF-1 levels acting on A*β* transport across the blood brain barrier. This point clearly needs further investigations.

Taken together, studies in humans have shown neuronal IR/IGF-1 resistance as being part of the pathogenesis of AD. However, there seems to be a dual role of IR/IGF-1 signaling in the pathogenesis of AD. Recent animal experiments indicate that central insulin and IGF-1 resistance is most likely a compensatory mechanism to *β* amyloid pathology to reduce A*β* toxicity and promote survival. But decreased IR/IGF-1 signaling causes tau hyperphosphorylation which is neurotoxic at least to certain extend. Experiments in *C. elegans* suggested that FoxO transcription factors might be involved in mediating the beneficial effects of reduced IR/IGF-1R signaling. However, studies in rodents favor that at least transgenically induced increased FoxO-mediated transcription might be harmful at least for AD pathology or AD-associated mortality, favoring a concept for the exigency of optimal balanced IR/IGF-1R → FoxO-mediated transcription under disease conditions.

## 9. Alternative Targets Regulated by Insulin/IGF-1 Signaling Possibly Involved in Aging and Pathogenesis of Dementia

However, FoxO-mediated transcription is not the only target of the IR/IGF-1R signaling cascade. Alternative effectors downstream the IR/IGF-1R have been suggested as possibly being involved in the pathogenesis of dementia or aging itself.

The PI3K pathway leads to activation of AKT. In addition to phosphorylation of FoxO, it regulates tuberin 2 (TSC-2). TSC-1 and -2 form a heterodimer harboring GTPase activity and inhibiting the GTPase RHEB (RAS homolog enriched in brain). Upon phosphorylation, the RHEB-GTP complex accumulates and leads to activation of mTOR (mammalian target of rapamycin) [146, 147]. Activated mTOR phosphorylates 4E-BP (4E binding protein) which then releases eIF4E (eukaryotic initiation factor 4E) promoting translation initiation. Furthermore, activated PDK1 and mTOR activate the S6 kinase (S6K). S6K phosphorylates eEF2 (eukaryotic elongation factor 2) kinase to release eEF2 which leads to initiation of elongation [148, 149]. Hence, the IR/IGF-1R signaling cascade increases protein synthesis in general. Recently, inhibition of mTOR using rapamycin has been shown to increase lifespan and age-dependent cognitive deficits in mice [150, 151]. At the moment, no data addressing the role of mTOR for the pathogenesis of AD are available, but this will be an interesting direction for further research.

The insulin/IGF-1 signaling cascade does not only regulate the PI3K pathway but also the MAP kinase (MAPK, mitogen-activated protein kinase) cascade. After activation of the insulin/IGF-1 signaling pathway, GRB-2 binds to phosphorylated IRS proteins [36]. After that, GRB-2 recruits son of sevenless (SOS), which is a GDP/GTP exchange factor. Then the activation of the small G-protein RAS results in association of c-raf leukemia viral oncogene (CRAF) to the membrane activating kinase (MAPK). Finally, the MAP kinases activate the extracellular signal-regulated kinase (ERK-1/-2) [46]. The activity of ERK-1/-2 has been shown to be involved in long-term potentiation and memory in the CNS [47]. Thus, the MAP-kinase pathway might be involved in certain aspects of the pathogenesis of dementia as well.

## 10. Conclusion

Obesity in midlife and type 2 diabetes are associated with an increased risk for vascular dementia and Alzheimer's disease. One common feature of obesity and type 2 diabetes is hyperinsulinemia leading to insulin desensitization. This has been identified at least as risk factor for cognitive decline. At least in AD, neuronal IR/IGF-1R signaling is severely impaired. Thus, reduced IR/IGF-1R signaling is part of the pathogenesis of AD. Interestingly, deletion of the IGF-1R in mouse models of AD leads to reduced mortality and decreased A*β* load. Furthermore, haploinsufficiency for the IGF-1R increased A*β* aggregation which is hypothesized to be a molecular mechanism of detoxification. Interestingly, neuron-specific deletion of the IR reduced the A*β* burden without influencing mortality. Thus, reduced IR/IGF-1R signaling observed in AD brains might be a compensatory mechanism protecting the brain against the toxic influence of chronic elevated insulin levels. Data obtained from *C. elegans* indicated that the FoxO transcription factors might mediate the beneficial effect induced via decreased IR/IGF-1 signaling. However, very recent data in rodents using transgenic expression of different FoxO variants nearly excludes FoxO1-induced transcription as mediators for these effects.
